# Bacterial extracellular vesicles exhibit distinct functional potential across biogeographic provinces of the South Pacific Ocean

**DOI:** 10.1093/ismejo/wrag171

**Published:** 2026-07-01

**Authors:** Eduard Fadeev, Neza Orel, Tinkara Tinta, Leila Afjehi-Sadat, Haoran Liu, Thomas J Browning, Zhongwei Yuan, Eric P Achterberg, Steven J Biller, Daniel J Sher, Gerhard J Herndl

**Affiliations:** Department of Functional and Evolutionary Ecology, Bio-Oceanography and Marine Biology Unit, University of Vienna, 1030 Vienna, Austria; Marine Biology Station Piran, National Institute of Biology, 6330 Piran, Slovenia; Marine Biology Station Piran, National Institute of Biology, 6330 Piran, Slovenia; Mass Spectrometry Unit, Research Support Facilities, University of Vienna, 1030 Vienna, Austria; GEOMAR Helmholtz Centre for Ocean Research, 24148 Kiel, Germany; GEOMAR Helmholtz Centre for Ocean Research, 24148 Kiel, Germany; GEOMAR Helmholtz Centre for Ocean Research, 24148 Kiel, Germany; GEOMAR Helmholtz Centre for Ocean Research, 24148 Kiel, Germany; Department of Biological Sciences, Wellesley College, 02481 Wellesley, United States; Department of Marine Biology, Leon H. Charney School of Marine Sciences, University of Haifa, 3103301 Haifa, Israel; Department of Functional and Evolutionary Ecology, Bio-Oceanography and Marine Biology Unit, University of Vienna, 1030 Vienna, Austria; Mass Spectrometry Unit, Research Support Facilities, University of Vienna, 1030 Vienna, Austria; NIOZ, Department of Marine Microbiology and Biogeochemistry, Royal Netherlands Institute for Sea Research, 1790 AB Den Burg, The Netherlands

**Keywords:** bacterioplankton, membrane vesicles, TonB-dependent receptors, siderophores, saccharide uptake system

## Abstract

Bacterial extracellular vesicles (BEVs) are nanoscale membranous structures released by diverse types of bacteria, and are capable of transporting and delivering biological compounds between cells. Experimental investigation of BEVs in laboratory model systems indicates that these nanoparticles may play a number of roles in the ecophysiology of marine bacterial communities, but their functional potential in the environment remains unclear. Here we describe the proteomic composition of BEV populations over more than 5000 nautical miles of surface waters in the South Pacific, linking BEV cargoes to the bacterial communities producing them. The presence of marine BEVs was consistently observed across a range of biogeochemical conditions, with an overall abundance comparable to that of bacterial cells (up to 10^8^ BEVs L^−1^). The protein cargo of marine BEVs, however, differed significantly among ocean regions. The BEV populations were enriched in carbohydrate transporters under phytoplankton bloom conditions, and contained iron uptake-related proteins in nutrient-limited waters. These data suggest that BEVs could enable cells to perform key extracellular functions in the marine environment. Our observations highlight the ubiquity of marine BEVs and biogeographic patterns in their ecological potential across oceanic scales.

## Introduction

Bacterial extracellular vesicles (BEVs) are nanoscale, membrane-bound structures typically ranging in diameter from 20 to 250 nm, which are released by bacterial cells. They can result from “blebbing” mechanisms in intact cells or be formed as a by-product of cell lysis. These different biogenesis mechanisms can influence BEV contents and, consequently, their functional potential [1]. Since their discovery in human-associated *Escherichia coli* in the 1960s [2], most studies of BEVs have examined their roles within laboratory model organisms, particularly host-associated bacteria in animal and plant systems. Such efforts have shown that BEVs can serve as a mechanism for removing harmful compounds from the cell, but can also transport various biological cargoes (e.g. nucleic acids and proteins) over long distances in a protected and locally concentrated manner [3]. One of the best-documented functions of BEVs in intercellular interactions is the transfer of DNA molecules [4, 5], which can facilitate the exchange of antibiotic resistance genes between different bacterial lineages [6], as well as the transfer of entire plasmids [7, 8], recently termed “vesiduction” [4]. BEVs can also play roles in bacterial quorum sensing [9, 10], biofilm formation [11], and contribute to microbial “warfare” by delivering compounds that kill competing bacteria [12, 13]. As BEVs can contain a variety of components released by bacterial cells, including enzymes, receptors, and transporters derived from cell membranes, they may interact with organic and inorganic chemicals in the environment [14, 15]. Such extracellular activities, including the enzymatic digestion or binding of various environmental compounds, are particularly important for bacteria living in aqueous environments [16, 17].

The ocean, where bacteria are the most abundant organisms [18], is a highly dynamic environment both physically and biochemically. The surface ocean is characterized by a depletion of bioavailable nutrients required for life in some vast areas (e.g. oligotrophic oceanic gyres) [19] and a high availability in others (e.g. coastal upwelling regions) [20, 21]. To survive in such ecosystems, marine bacteria have developed a wide range of ecophysiological capabilities [22–24], and implement highly diverse nutrient and energy utilization strategies [25–27]. In the interaction between marine bacteria and their oligotrophic environment, high cellular surface area to volume ratios facilitate efficient nutrient uptake [28, 29]. In this sense, the release of marine BEVs by bacterial cells can be conceptualized as an expansion of the cell surface which disperses through the aqueous medium [1], randomly encountering various components of the marine environment. Laboratory observations of several isolates of globally abundant marine bacteria suggest that BEVs may mediate specific extracellular functions in the ecophysiology of these bacteria, such as binding nutrients (e.g. iron or phosphate) and delivering them in concentrated form to bacterial cells [30–35]. However, despite the potential importance of BEVs for marine bacteria, and apparent ubiquity in the marine environment [36, 37], our understanding of their biogeographic diversity across large environmental gradients is highly limited [35].

To address this knowledge gap, we conducted a spatially extensive sampling to examine the abundance and protein cargo diversity of BEVs across the South Pacific Ocean. We asked how the molecular cargo of marine BEV populations varies in context with their local microbial and biogeochemical environment, finding that BEVs may play distinct functions for different microbial populations. Our observations across large oceanic regions reinforce the emerging picture that important extracellular bacterial functions may be mediated by BEVs and can inform future efforts to test specific hypotheses about their ecological roles.

## Materials and methods

### Sample collection

**Figure 1:**
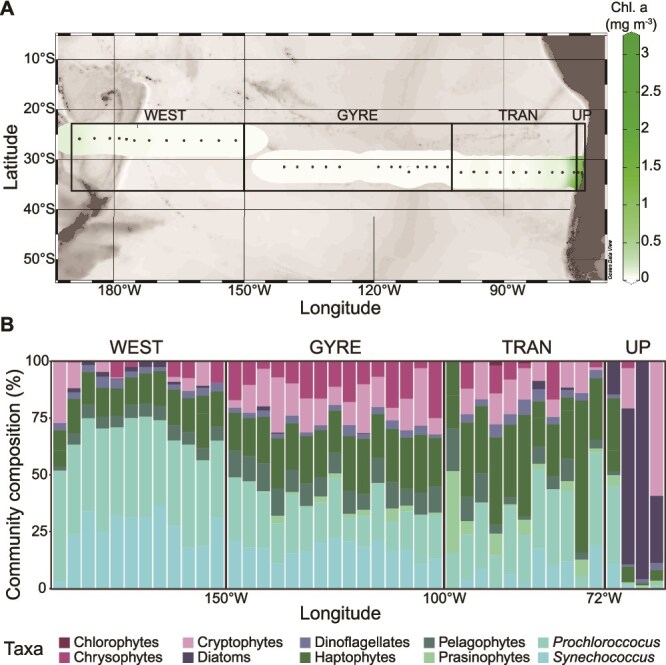
Diverse biogeochemical regimes encountered across the South Pacific Ocean. (A) Spatial grouping of the sampling stations according to four oceanic provinces: “UP”—Chilean coast upwelling zone; “TRAN”—transition zone between the upwelling and the subtropical GYRE; “GYRE”—South Pacific subtropical gyre; “WEST”—westernmost region. (B) The composition of the surface water phytoplankton at each station, estimated on the basis of diagnostic pigments.

The oceanographic sampling was conducted onboard RV *Sonne* (cruise SO289 representing section GP21 of the GEOTRACES program) from 18 February to 8 April 2022 (Fig. 1). Water samples with a total volume of 60 L were taken from a depth of ~6 m at each sampling station using the onboard water pump. To remove particulate matter and eukaryotic cells, water samples were pre-filtered at low pressure using a 3 μm pore size membrane filter (Merck Millipore, MA, USA). From the pre-filtered water sample, 1 ml was preserved with 4% glutaraldehyde (final concentration) and stored at −80°C until flow cytometric analysis (analysis described in Supplementary Text). The pre-filtered seawater sample was then filtered through a 142 mm diameter polycarbonate filter with a pore size of 0.22 μm (Merck Millipore, MA, USA), which were then stored at −80°C and later used for molecular analyses of the bacterial communities. The filtrate (<0.22 μm) was concentrated approx. 120 times using a Vivaflow tangential flow filtration system (Sartorius, Germany) with a 100 kDa cut-off to 0.5 L and stored at −20°C until further BEVs purification in the laboratory. Additional bulk seawater samples of 2–4 L were collected for diagnostic phytoplankton pigments analysis following the procedure described elsewhere [19].

### Bacterial extracellular vesicles isolation and quantification

A sub-sample of 100 ml of concentrated seawater was further concentrated about 30 times to a final volume of 3 ml using Vivaspin centrifugal filter units (Sartorius, Germany) with a 100 kDa cut-off. The entire concentrate was then loaded onto an iodixanol density gradient following a published protocol [38], and centrifuged at 100 000× *g* at 4°C for 6 h. In the 15%–30% and 35%–40% fractions, the iodixanol solution was exchanged twice with 1 × PBS using Vivaspin centrifugal filter units with a 100 kDa cut-off at 2000× *g*, and resuspended in 1 ml 1 × PBS. From each purified fraction, 800 μL was used for molecular analyses and 200 μL for quantification of BEVs.

The abundance of BEVs was measured by nanoparticle tracking analysis (NTA) using a NanoSight NS300 instrument (Malvern Panalytycal, UK) equipped with a 488 nm laser. Each sample was injected using a syringe pump with a flow speed of 100 μL min^−1^, and five videos of 60 s were recorded. Videos were analyzed using NanoSight NTA software v3.2. Out of the five technical replicates, a consistent triplicate was selected using Kolmogorov–Smirnov test (i.e. triplicate with similar size distribution pattern). All particles in the size of 20–250 nm in both the 20%–30% and 30%–40% fractions were included in the final abundance estimates. To estimate the background, we quantified nanoparticles in Optiprep media that was subjected to the same washing steps with 1 × PBS as the BEVs samples, yielding concentrations of about 10^5^ particles ml^−1^, with most particles being below 50 nm in size.

### Metagenomics analysis

One-third of the polycarbonate filters with a pore size of 0.22 μm was used for the extraction of genomic DNA using the DNeasy PowerWater Kit (QIAGEN N.V., Germany) according to the manufacturer’s recommendations. The extracted DNA was subjected to enzymatic shearing and then high-throughput sequencing, performed on two lanes of the NovaSeq S4 System (Illumina, Inc., CA, USA) at the Vienna Biocenter Core Facilities (https://www.viennabiocenter.org/vbcf/next-generation-sequencing/).

The sequences from all samples were analyzed using the snakemake [39] metagenomic workflow in Anvi’o v8 [40], with recommended parameters. Briefly, the raw FASTQ files were subjected to quality control by “illumina-utils” v2.13 [41] and taxonomic classification using KrakenUniq v1.0.4 [42] and analyzed using R package “pavian” v1.2.1 [43]. Then the reads were assembled using MEGAHIT v1.2.9 [44] and the gene calling was performed using Prodigal v2.6.3 [45]. To construct the reference database for downstream metaproteomic analysis, identified protein coding genes were de-replicated using CD-HIT v4.6.881 [46], excluding sequences with less than 60 amino acids.

Taxonomic affiliation of the protein coding genes was estimated on a contig basis using Contig Annotation Tool [47] against the GTDB database (date: 20 November 2023), complemented with sequence based annotation using Kaiju v1.9.2 [48] against the NCBI nr database (date: 25 August 2024). Functional annotation of the protein coding genes was carried out against InterPro v98 [49], NCBIfam v17.0 [50], and Pfam v37 [51] databases using InterProScan v5.66 [52]. The unidentified proteins sequences with no hits, were assigned to their taxonomy and function using BLASTp [53] best-hit approach against NCBI RefSeq database (date: 27 January 2025). Protein sub-cellular localization predictions were established using functional annotation combined with signal peptides identification using SignalP v6 [54], transmembrane topology prediction using Phobius [55], and sub-cellular localization predictions using DeepLocPro v1.0 [56]. Porins and TBDRs were further classified using PSIBLAST [56] against the Transporter Classification Database (TCDB) [57], best hit was defined based on identity >30%, e-value <0.001 and bit score > 50. The potential ligands of the TBDRs were inferred from homology comparison to previously characterized and curated transporters in TCDB as well as through manual exploration of their genomic contexts in the metagenomic contigs.

### Metaproteomics analysis

We used the remaining two-thirds of the 0.22 μm pore-size polycarbonate filters for extraction of cellular proteins following an established protocol [58]. A brief description of the protein extraction procedures and the mass spectrometry analysis can be found in the Supplementary Text.

The MS raw files of proteins extracted from the bacterial cells and the BEVs were processed using the Spectrum Files RC node in Proteome Discoverer v2.2.0.338 (ThermoFisher Scientific, MA, USA) and searched using SEQUEST-HT against the bacterial protein coding genes from the co-assembled metagenome. Search parameters were as follows: enzyme—trypsin, max. missed cleavages—2, min. peptide length 6, precursor mass tolerance—9 ppm, fragment mass tolerance—0.05 Da, static modifications—carbamidomethyl (Cys), dynamic modifications—oxidation (Met). Percolator parameters were as follows: max. delta Cn: 0.05, max. rank: 0, false discovery rate (calculated by automatic decoy searches) 0.05, validation based on q-value. Abundance of each protein was estimated by summing the abundance of unique and razor peptides with at least 2 Peptide Spectrum Matches. Only proteins with at least one unique peptide and at least two peptides in total (unique and/or razor peptides) were included in the statistical analyses.

### Statistical analyses

Statistical analyses were done in R v4.4.2 (R Core Team 2022) using RStudio v2024.09.1 (RStudio Team 2019). Prior to statistical analyses, the protein abundance matrix was log_2_-transformed and median-centered. Protein enrichment analysis was performed using the R package “DEqMS” v1.22.0 [59]. Only those proteins were included that were observed in at least two samples in each fraction. The criteria for a significantly enriched protein were an absolute log_2_ fold change >1, adjusted *P* value <.1, and presence in at least two samples in each group (e.g. fraction or region). The enrichment analysis of non-cytoplasmic proteins included only those with a high-confidence prediction of sub-cellular localization, based on a combination of the presence of a signal peptide and functional annotation.

## Results and discussion

### Distinct biogeochemical conditions across the South Pacific Ocean

To investigate the potential ecophysiological functions of BEVs in marine bacterial communities we sampled across diverse environmental conditions, varying in nutrient limitations and phytoplankton growth conditions. We collected surface water (5–6 m depth) at 20 stations along a basin-scale transect that spanned the South Pacific Ocean (Fig. 1; Table S1). A detailed physicochemical characterization of this transect showed that field sampling was carried out across four oceanic provinces [60, 61]. Briefly, the easternmost region was the Chilean coastal upwelling zone (UP), characterized by elevated nitrate and phosphate concentrations, but depleted in dissolved iron. In this area there were elevated chlorophyll-a concentrations (~3 mg m^−3^; Fig. S1), with the phytoplankton community mostly composed of diatoms (Fig. 1). The transition zone between the upwelling and the subtropical gyre (TRAN) was characterized by elevated concentrations of phosphate and dissolved iron, but was depleted in nitrate (Fig. S1). This area also had relatively elevated chlorophyll-a concentrations (0.04–0.26 mg m^−3^), with mixed phytoplankton communities. The center of the subtropical gyre (GYRE) was characterized by very low nitrate concentrations and a declining spatial gradient from east to west of phosphate concentrations, with strongly depleted chlorophyll-a concentrations (< 0.03 mg m^−3^; Fig. S1). The westernmost region (WEST) was defined as the area of the subtropical gyre with the lowest phosphate concentrations and higher (compared to GYRE) chlorophyll-a concentrations (0.03–0.14 mg m^−3^; Fig. S1), with the phytoplankton comprising predominantly cyanobacteria.

**Figure 2:**
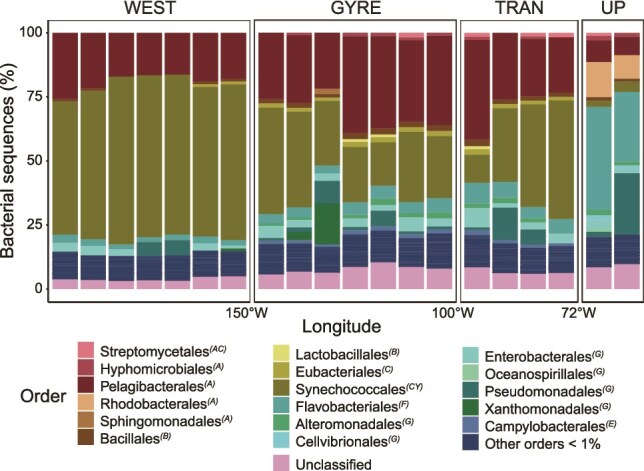
Bacterial community composition based on taxonomic classification of metagenomic sequences. The taxonomic class of each order is given in parentheses: “AC”—Actinomycetes, “A”—Alphaproteobacteria, “B”—Bacilli, “C”—Clostridia, “CY”—Cyanophyceae, “F”—Flavobacteriia, “G”—Gammaproteobacteria, “E”—Epsilonproteobacteria. “Other orders < 1%”—refers to taxonomic orders that comprised <1% of the sequences; “unclassified”—refers to bacterial sequences with unknown classification on an order level. All samples contained 2–5 × 10^8^ classified sequences.

**Figure 3:**
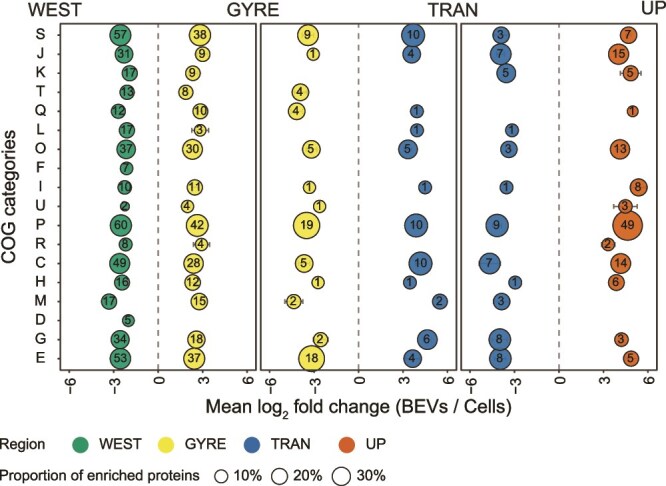
Overview of significantly enriched proteins in the cellular fraction between bacterial communities in different regions. Proteins are group according to cluster of orthologous genes (COG) categories. The numbers represent the absolute number of enriched proteins in each category, and the size of the circle represents it proportion among all enriched significantly proteins in the region. The COG categories accessions correspond to: S: Function unknown, J: Translation, ribosomal structure and biogenesis, K: Transcription, T: Signal transduction mechanisms, Q: Secondary metabolites biosynthesis, transport and catabolism, L: Replication, recombination and repair, O: Posttranslational modification, protein turnover, chaperones, F: Nucleotide transport and metabolism, I: Lipid transport and metabolism, U: Intracellular trafficking, secretion, and vesicular transport, P: Inorganic ion transport and metabolism, R: General function prediction only, C: Energy production and conversion, H: Coenzyme transport and metabolism, M: Cell wall/membrane/envelope biogenesis, D: Cell cycle control, cell division, chromosome partitioning, G: Carbohydrate transport and metabolism, E: Amino acid transport and metabolism.

We found that bacterial abundance varied across the biogeochemical provinces (Table S1), and the taxonomic composition of the bacterial communities (Fig. 2) also differed significantly between them (PERMANOVA test; *F*_3,14_ = 21.2, *R*^2^ = 0.82, *P* < .01). To investigate the physiological and metabolic processes expressed within the bacterial communities, we characterized the metaproteome of the bacterial cells, identifying between 2270 and 5734 cellular proteins at each station (Table S2). The overall composition of bacterial proteins in the cellular fraction significantly varied between the four regions (PERMANOVA test; *F*_3,16_ = 3.2, *R*^2^ = 0.37, *P* < .01; Fig. S2) and further differed between each pair of adjacent provinces (Fig. 3 and Table S3). In the upwelling zone (UP), where bacterial cell abundance was the highest (6–22 × 10^8^ cells L^−1^), we identified a total of 133 significantly enriched proteins (Table S3, log2 fold change >1, adjusted *P* value <.1). The taxonomic lineages that comprised the most enriched proteins were the orders *Cellvibrionales* and *Flavobacteriales* (38% and 24% of all enriched proteins, respectively), both of which are typically active during phytoplankton blooms [62, 63]. The enriched bacterial proteins in this region were associated with β-oxidation (e.g. acyl-CoA dehydrogenases), energy production (e.g. ATP synthase subunits), various ribosomal proteins, and TonB-dependent uptake systems (Fig. 3). In the transition zone, bacterial abundance reached 0.6–2.4 × 10^8^ cells L^−1^, with the *Cyanobacteria* comprising a major fraction of the bacterial community (Table S1). In this region, we identified a total of 109 significantly enriched proteins, 45% of which were cyanobacterial proteins (Table S3). These enriched proteins were associated with photosynthetic activity (e.g. phycobilisome-related proteins) and cellular activity (e.g. RNA polymerases). The subtropical gyre had the lowest bacterial cell abundances (0.08–1.2 × 10^8^ cells L^−1^). We identified a total of 326 significantly enriched proteins in this region (Table S3), with the order *Pelagibacterales* and SAR86 clade comprising 22% and 17% of these proteins, respectively. These proteins were associated with cellular metabolism (e.g. various dehydrogenases) as well as nutrient uptake-related proteins (e.g. various transporters and solute binding proteins). The *Pelagibacterales* are the most abundant bacteria in the surface ocean, predominantly in nutrient-limited conditions [64], and together with SAR86 they have been previously shown to dominate the bacterial communities of the ultra-oligotrophic South Pacific subtropical gyre [65, 66]. In the westernmost region (WEST), cell abundances were significantly higher than in the subtropical gyre (0.7–1.6 × 10^8^ cells L^−1^), with a predominance of cyanobacteria. We identified a total of 449 significantly enriched bacterial proteins in this region, almost half of which were cyanobacterial proteins (Table S3). The predicted functional categories of the enriched cyanobacterial proteins indicated cellular (e.g. ribosomal proteins and cell division-related proteins) and photosynthetic activity (e.g. numerous photosystem-related proteins).

Together with the ecological and biogeochemical characteristics of the different regions, this analysis indicates a heterotrophic bacterial response to the availability of organic carbon as a result of the diatom bloom in the upwelling zone, cyanobacterial activity in the transitional zone (likely stimulated by a nutrient influx from a returning lateral current) [60], strongly oligotrophic conditions in the subtropical gyre characterized by dominance of scavenging oligotrophic bacteria, and an elevated abundance and activity of *Prochlorococcaceae* in the westernmost region, which may have been partly facilitated by input from a large volcanic eruption in the region two months prior to sampling [67].

### Abundance, characteristics, and main producers of marine bacterial extracellular vesicles

**Figure 4:**
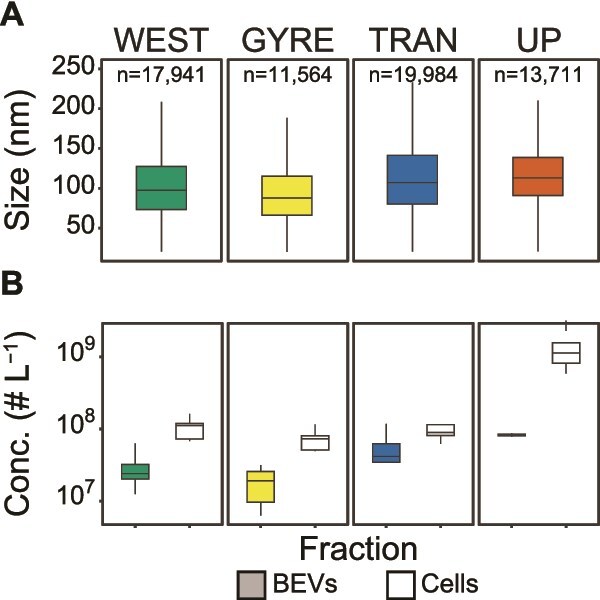
Physical characteristics of marine BEV-like structures. (A) Estimated diameter distribution of all BEV-like particles in each region. (B) Bacterial cell and BEVs abundances, estimated using flow cytometry and NTA, respectively.

To investigate the abundance and diversity of marine BEVs, we sampled the extracellular size fraction corresponding to BEV-like structures (100 kDa–0.22 μm) across all stations using size fractionated filtration. The size distribution of marine viruses overlaps with that of BEVs [68–70], so we used density gradient separation to further enrich our samples for BEVs in the laboratory (see Materials and Methods). The presence of BEV-like structures in the selected density fraction was confirmed by observing membrane-bound structures using transmission electron microscopy (Fig. S3). At least 50% of the purified BEVs were between 70 and 130 nm in diameter (Fig. 4), matching the size distributions previously observed in cultures of marine bacteria [33, 35]. Their abundance was lower than the abundance of bacterial cells (Fig. 4). This is probably an underestimate, given that the density gradient separation resulted in a yield of only around 4% of the total nanoparticles in the seawater (Fig. S4). This loss of biomass is likely due to the thorough washing and concentration steps during the further purification of BEVs in the laboratory, compared to the bulk nanoparticles samples collected on board (see Material and Methods).

We characterized the bulk protein content in the BEV-enriched density fraction (see Materials and Methods) and identified 235–678 proteins at each station (Table S1). Of these, only 3–37 proteins were related to viruses in terms of taxonomy or function (e.g. capsid or tail sheath). As the metagenomic dataset used for the proteomic analysis was generated using DNA extracted from the cellular fraction (>0.22 μm), only 4% of the protein-coding genes in the dataset were associated with viruses. Therefore, it is possible that the viral proteome is underrepresented in our analysis, resulting in some viral proteins remaining undetected. The viral proteins that were identified accounted for <1% of each sample (see Fig. S5), which suggests that BEVs were enriched to some extent compared to other types of protein-containing marine nanoparticle in the targeted density fraction. We found that 43%–61% of total number of proteins observed in the BEVs were also found in the corresponding cellular fraction at each station, but the composition of these proteins significantly varied between the two fractions (PERMANOVA test; *F*_1,38_ = 9.1, *R*^2^ = 0.19, *P* < .01). This indicates that the BEV-associated proteins represent a distinct subset of the bacterial cellular proteome. To explore the differences between the cellular and BEV-associated proteomes, we performed an enrichment analysis (Table S4). As the biogeochemical conditions sampled in this study across the different regions shape the bacterial communities (Fig. 2), they are also likely affecting BEV populations and functions [1, 17, 70, 71]. To account for these differences, we examined the proteins enriched in BEVs separately for each oceanic province. As only two stations were sampled in the upwelling region, this region was excluded from further analysis. We found a total of 201 proteins significantly enriched in BEVs compared to 284 proteins enriched in bacterial cells (absolute log_2_ fold difference > 1, adjusted *P* < .1, observed in at least two samples in each region).

**Figure 5:**
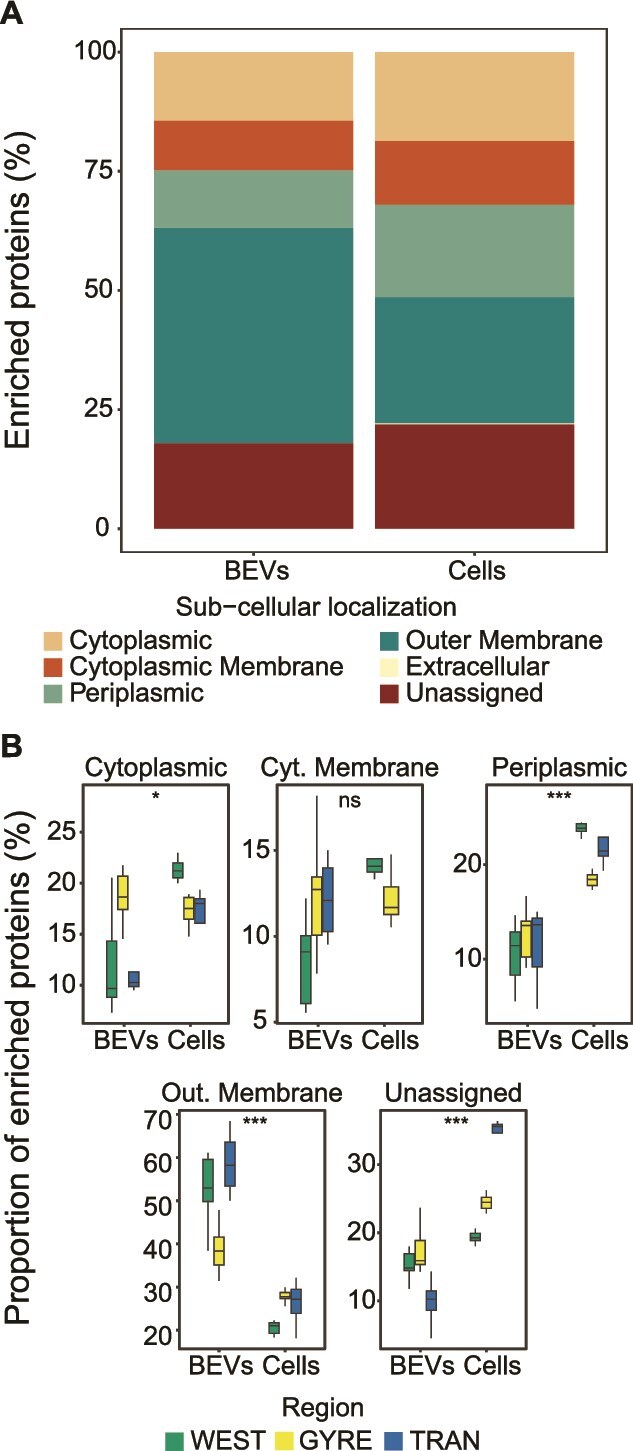
Overview of significantly enriched proteins in BEVs and cellular fractions. (A) The bar plots represent the composition of all significantly enriched proteins according to their estimated sub-cellular localization. (B) Proportional distribution of enriched proteins of each sub-cellular source in each sample, grouped by regions and fractions. Significance of variation in proportions between fractions was carried out using pairwise Wilcoxon signed-rank test (^***^)—adjusted *P* < .001, (^*^)—adjusted *P* < .05, (ns)—adjusted *P* > .1.

Bacterial cells produce BEVs through various processes, such as membrane blebbing and lysis, which facilitate the export of proteins of various cellular origins [1, 17, 71, 72]. The enriched proteins in our dataset revealed a heterogenous composition of sub-cellular origins (Fig. 5). Outer membrane proteins were found to be more abundant in the BEVs than in the cellular fraction. However, there was also a notable presence of cytoplasmic proteins in the marine BEVs. This suggests that the BEVs in our bulk samples may have derived from a variety of formation processes. While these observations could have been influenced by the naturally higher surface-to-volume ratio of BEVs compared to cells, as well as various methodological biases, such as the enrichment of specific types of BEVs, they suggest that marine BEVs contain a highly diverse range of proteins from different cellular sources, which affects their functional potential.

**Figure 6:**
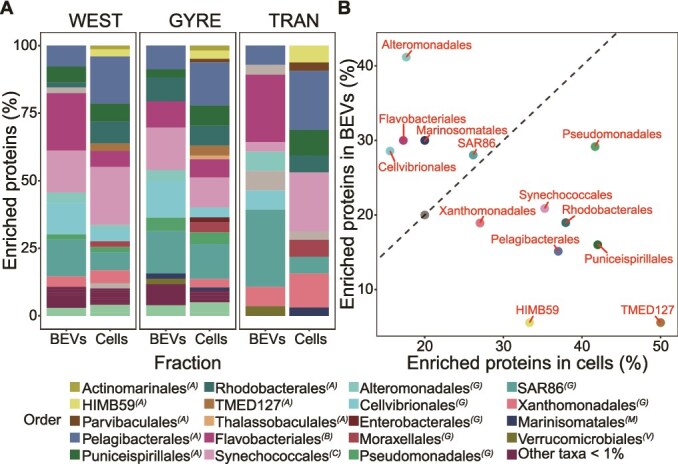
Taxonomic classification of significantly enriched proteins in BEVs and cellular fraction in each taxonomic order. (A) Proportional taxonomic composition of significantly enriched proteins. (B) The graph shows the percentage of cell-enriched (x-axis) and BEV-enriched (y-axis) proteins identified for different taxonomic orders. The dashed line represents a theoretical ratio of 1:1 between the number of proteins enriched in each fraction.

Studies of laboratory cultures indicate that many abundant groups of marine bacteria exhibit significant variations in BEV production rates [35]. Our protein enrichment analyses from field samples revealed differentially abundant proteins associated with 27 different bacterial orders. In the cellular fractions, the two taxonomic lineages with the largest proportion of enriched proteins were *Cyanobacteria* (mostly *Prochlorococcus*) and *Pelagibacterales*, comprising 16%–22% and 11%–22%, respectively, of all enriched proteins. In contrast, among the BEVs fraction the taxonomic lineage with the largest proportion of enriched proteins was the gammaproteobacterial clade SAR86 with 14%–29% of all enriched proteins, followed by the order *Flavobacteriales* with 10%–25% of all enriched proteins (Fig. 6). The ratio of enriched proteins between cellular and BEVs fractions of these lineages showed a clear tendency towards the latter. These observations could indicate that BEVs from these lineages contain more proteins on average than those from Alphaproteobacteria and cyanobacteria. This is consistent with previous culture-based observations of numerous BEV-enriched proteins in *Alteromonadales* [32], endosymbiotic *Cellvibrionales* [73], and other marine Gammaproteobacteria [31, 74]. Alternatively, based on the assumption that the number of enriched proteins reflects to some extent the abundance of BEVs, these results are consistent with the previously raised hypothesis that fast-growing heterotrophic marine bacteria contribute more to the BEV population than slow growing ones [74–77].

### Marine bacterial extracellular vesicles as passive scavengers of iron

External conditions have been shown to have an impact on the release of BEVs by bacterial cells, potentially leading to some degree of preferential protein packaging [75–78]. To test the hypothesis that specific functions are enriched in marine BEVs in response to environmental conditions, we compared the proteins present in BEVs with those in cells, focusing specifically on the non-cytoplasmic proteins identified in our dataset (Table S5). This is because, as with bacterial cells, it is expected that functional interactions between a BEV and its environment will be predominantly carried out by outer-membrane and periplasmic proteins [78]. We were unable to identify in our dataset any significant links between specific parameters and individual BEV-associated proteins, which is likely due to the strong correlations between the measured environmental parameters (Fig. S1). However, an enrichment analysis according to the three provinces, which had distinct combined ecological conditions, revealed different enrichment dynamics for functionally distinct groups of proteins in BEVs (Table S5). We found that in all three provinces the largest group of proteins significantly enriched in marine BEVs was TonB-dependent receptors (TBDRs), comprising 91 different proteins, compared to 52 in the cellular fraction. The majority of the BEV-enriched TBDRs belonged to the one-component system of the outer-membrane receptor family that performs both binding and transport of the substrate through the outer membrane (Fig. S6). These TBDRs are involved in cellular uptake of a wide range of substrates, such as iron and iron-containing molecules, as well as vitamins and various organic compounds [79]. We found that the taxonomic lineage with the largest number of BEV-enriched TBDRs was the SAR86 clade (class Gammaproteobacteria). Previous genomic characterization of this lineage revealed that, despite their streamlined genome, they possess a disproportionately large number of TBDRs [80], and are often found in nutrient depleted waters, such as the South Pacific Ocean [66].

It has been demonstrated that iron limitation can lead to enhanced BEVs production in non-marine bacteria [81] and preferential packaging of iron-related transporters into BEVs has been proposed to facilitate iron accumulation [78, 82, 83]. In our study, iron was highly depleted in surface waters across most of the transect [60, 61]. Although the exact ligand for most of the TBDRs in our dataset remained unidentified, we found 11 different siderophore-related TBDRs enriched in BEVs, compared to only four in the cellular fraction. Siderophores are small molecules that bind strongly iron in the environment and are subsequently acquired by bacterial cells through specialized TBDRs [84–86]. To actively transport molecules across the outer membrane, such TBDRs require proton motive force from the inner membrane [87]. Because BEVs are unlikely to have a proton motive force, they can still bind the substrates, as was recently demonstrated in a culture of a pathogenic Gammaproteobacterium [88]. Taking this into account, we propose that marine BEVs may “collect” iron-rich molecules in the environment, and therefore can be considered nanoscale “hotspots” of bioavailable iron which could potentially provide a relatively concentrated source for marine bacteria. Taken together, these findings suggest that BEVs might play a role in extracellular scavenging of iron by marine bacteria.

### Marine bacterial extracellular vesicles as mediators of carbohydrate utilization

**Figure 7:**
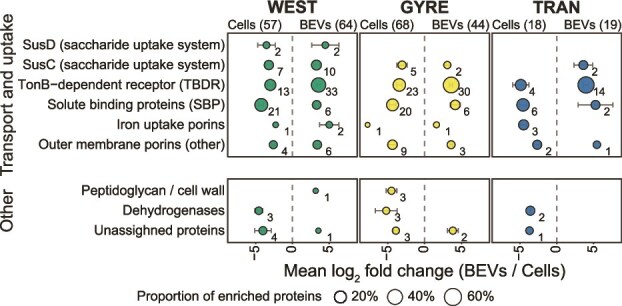
Non-cytoplasmic proteins significantly enriched in BEVs and cellular fractions in different regions. The mean log₂ fold difference was calculated for each protein group in each fraction. Positive log_2_ fold difference represents significant enrichment in the BEVs fraction, negative mean log_2_ fold difference represents significant enrichment in the cellular fraction. The total number of enriched proteins in the BEVs and the cellular fractions is provided in brackets. The proteins were grouped according to their proposed functions in BEVs. For visualization purposes, only functional groups containing at least two enriched proteins in at least one region were included.

The second largest TBDR-related functional group enriched in BEVs was linked to the unique SusC-like protein family (Fig. 7), which play an important role in carbohydrate utilization by marine Flavobacteria (i.e. order *Flavobacteriales*). Flavobacteria efficiently utilize various sugars using a two component SusCD system, where SusC-like proteins are integral outer-membrane transporters and SusD-like lipoproteins facilitate extracellular high-affinity binding of the sugars [89]. We found a total of 13 SusC-like proteins enriched in BEVs, compared to 11 SusC-like proteins enriched in the cellular fraction, as well as 2 SusD-like lipoproteins enriched in both fractions (Table S5). The strongest enrichment of SusCD proteins was observed in the TRAN and WEST regions, where an elevated primary production activity was observed. Presence of SusCD proteins on BEVs was previously observed in an isolate of marine *Flavobacteriales* (*Formosa* spp*.*), which appear to have developed means of producing BEVs that are found in chains attached to the cell [89]. Chains of such outer membrane-attached BEVs significantly increase the surface area through which the cell interacts with its environment, facilitating the accumulation of elements necessary for life in the vicinity of the cell and their subsequent uptake. It is likely that our pre-filtration step, which aimed to remove the particulate size fraction, disrupted any such structures present in the sample, and parts of these structures may have ended up in the bulk BEVs fraction.

Recent observations have shown that gut bacteria can actively alter the enzyme content of BEVs to optimize carbohydrate utilization [90]. Trapping carbohydrate molecules on the BEV membrane (or BEVs getting attached to a large carbohydrate) may facilitate their efficient digestion by hydrolytic enzymes in a manner similar to that observed for cellular outer membranes [31, 32, 90, 91]. Even though we lack such a mechanistic understanding for marine BEVs, the presence of hydrolytic enzymes associated with BEVs and their activity has been observed in several isolates of heterotrophic marine bacteria [31, 32, 91, 92]. We did not identify enrichment of hydrolytic enzymes in BEVs, due to their low representation in the proteomic samples (only 6 out of 13 629 proteins). Nevertheless, the association of enzymes with marine BEVs is conceptually advantageous because it enables them to be delivered to the substrate in a more protected manner than if they were dispersed freely in the water column. This may also facilitate more efficient degradation in combination with SusC- and SusD-like proteins [93]. When considered alongside other BEV-enriched proteins (Table S5), our observations on marine BEVs are in line with previous findings regarding the composition of BEVs in isolates of marine *Flavobacteriales* and *Alteromonadales* [93]. In these studies, it was suggested that BEVs play a role in carbohydrate utilization, based on their transporter and hydrolytic enzyme content. Previous work has shown that marine bacteria release hydrolytic enzymes into the extracellular milieu to facilitate carbohydrate utilization during periods of increased phytoplankton activity [94]—a phenomenon observed in the transitional zone and the western regions of the transect. This suggests that some of the secretion occurs via marine BEVs, potentially affecting degradation rates or access to the products.

### Marine bacterial extracellular vesicles as extracellular reserves of bio-available nutrients

The surface waters of the South Pacific subtropical gyre are one of the most oligotrophic regions in the global ocean [95]. Among the bacterial lineages that successfully inhabit such limiting environment are members of the order *Pelagibacterales* (also known as SAR11 clade). Their small cell size and large periplasm, which contains many substrate-binding proteins (SBPs) with exceptionally high affinities [96, 97], facilitates uptake of soluble substrates (e.g. phosphate, sulphate, amino acids, and sugars) from the environment [98]. This enables SAR11 to dominate the bacterial communities in the ocean [98] and thrive in nutrient-depleted marine ecosystems [99]. In BEVs of the GYRE and WEST enrichment we observed 13 different periplasmic SBPs, linked to the uptake of various substrates, such as amino acids and glycerol-3-phosphate (Fig. 7). Most of the BEV-enriched SBPs were taxonomically affiliated to *Pelagibacterales* (Table S5). We also observed enrichment in BEVs of 12 different outer-membrane porins, taxonomically affiliated mostly to *Cyanobacteria* and *Pelagibacterales*. We propose that porins form channels that allow small molecules to diffuse passively into the BEVs, as they would in intact cells. They become trapped there by binding to SBPs, making the BEVs a potential reservoirs for bio-available nutrients.

### Marine bacterial extracellular vesicles production—selfish trait or public good

Our data suggest that marine BEVs are produced by organisms that exhibiting both, planktonic (i.e. free-living) and a particle-associated (including phytoplankton) lifestyles [100, 101]. In contrast to the relatively confined particle-associated environments (e.g. biofilms), it is unlikely that BEVs released by a planktonic cell directly into seawater will be encountered again by the same, or a highly similar, bacterial cell [102, 103]. This led to the initial hypothesis that marine BEVs likely serve to dispose harmful elements or act as a defense mechanism against viral infection [82]. However, it has been shown that in some cases the uptake of BEVs only occurs between closely related bacterial lineages [82], suggesting that there may be mechanisms that bias interactions between BEVs and recipient cells [104]. Thus, BEV production could represent a mechanism through which cells compete for nutrients in the marine environment, potentially favoring only phylogenetically closely related populations.

The environmental observations of natural bacterial communities in this study, alongside previous observations of marine bacterial cultures, may also suggest other scenarios. One alternative explanation is that marine BEVs fulfill their intended function while remaining within the diffusive boundary layer [105]. Another possible scenario is that the BEVs remain attached to the cell, e.g. in the form of cellular extensions. Taking into account the potential stability of marine BEVs for at least a number of days [33], they may continue to perform their functions even after leaving the vicinity of the producing cell. Therefore, whether separated from cells or released directly into the environment, marine BEVs could also be considered a “public good” that meets the various needs of the bacterial community.

## Conclusions

Here we explored BEVs contents within highly diverse natural marine bacterial communities on an extensive oceanic scale in the South Pacific. We found that marine BEVs are ubiquitous in surface seawater and possess a substantial degree of protein diversity. The enrichment of functionally specific proteins in BEVs appears to be linked to the ecological conditions experienced by the local bacterial community. This suggests that BEVs may contribute to marine biogeochemistry and the metabolism of microbial communities. These findings reinforce the emerging hypothesis that BEVs play a role in nutrient and energy scavenging in the marine environment. Our study provides initial observations on the functional potential of marine BEVs, such as their ability to perform local enrichment of molecules necessary for life, which will motivate future experimental investigations into the underlying mechanisms. To develop a better understanding of the role of BEVs in the ecophysiology of marine bacteria, research into their uptake dynamics is needed. This is a significant knowledge gap regarding the relationship between BEVs and bacterial cells, which could help us understand whether BEVs are a “public good” or a competitive feature of marine bacterial communities. Based on the extensive oceanographic observations presented in this study, the next stage of the research requires the quantification of the contribution of BEVs to the distribution of essential life-sustaining elements in the marine environment. This will shed light on their importance for microbial processes in the ocean.

## Supplementary Material

Supplementary_material_wrag171

## Data Availability

The metagenomic raw sequences are available from the European Nucleotide Archive (ENA) at EMBL-EBI under Project accession number PRJEB88044. The mass spectrometry proteomics data can be obtained from the ProteomeXchange repository with the dataset identifier PXD062682. Scripts for data processing and statistical analyses, as well as raw flow cytometry and NTA files, can be accessed via Zenodo (https://www.zenodo.org/) under doi: 10.5281/zenodo.15772522.
