# Supplementary material for: Bacterial extracellular vesicles exhibit distinct functional potential across biogeographic provinces of the South Pacific Ocean
Source: ISME J. 2026 Jul 1;20(1):wrag171. doi: 10.1093/ismejo/wrag171 (PMC13398704; doi:10.1093/ismejo/wrag171)
Supplement: Supplementary_material_wrag171 [file supplementary_material_wrag171.zip › Marine_BEVs_supp_text_090626_wrag171.docx]

**Supporting Information for**

Bacterial extracellular vesicles exhibit distinct functional potential across the biogeographic provinces of the South Pacific Ocean

Eduard Fadeev, Neza Orel, Tinkara Tinta, Leila Afjehi-Sadat, Haoran Liu, Thomas J. Browning, Zhongwei Yuan, Eric P. Achterberg, Steven J. Biller, Daniel J. Sher, and Gerhard J. Herndl

Corresponding author: Eduard Fadeev

**Email:**  [eduard.fadeev@univie.ac.at](mailto:eduard.fadeev@univie.ac.at)

**This PDF file includes:**

Supporting text

Figures S1 to S5

Legends for Datasets S1 to S5

SI References

Supplemental Materials and Methods Text

Phytoplankton pigment analysis

Seawater samples (2-4 L) for diagnostic phytoplankton pigments were filtered onto MF300 glass fiber filters (ThermoFisher Scientific, MA, USA) and stored at -80°C until analysis. Pigment extraction and analysis followed the procedure described elsewhere [1]. Briefly, upon return to the land-based laboratory, pigments were extracted in 90% acetone by homogenization of the filters using glass beads in a cell mill, centrifuged (10 min, 5200 rpm, 4°C). The supernatant was filtered through 0.2 μm PTFE filters (VWR, PA, USA) and subsequently analyzed by reverse-phase high performance liquid chromatography (HPLC) Dionex UltiMate 3000 LC system (ThermoFisher Scientific, MA, USA)[2]. Diagnostic pigments were converted to estimated contributions of different phytoplankton types using CHEMTAX [3], with initial pigment ratios [4]. Total chlorophyll-*a* derived from HPLC include both chlorophyll-*a* and divinyl chlorophyll-*a*, and is referred to as chlorophyll-*a* throughout this study.

Bacterial cell counts

A subsample of 100 μL of seawater from each sampling stations was stained with 1×SYBR Green I (Thermo Fisher Scientific, MA, USA) for 15 min at room temperature and then quantified using FACSAria Flow Cytometer (BD Biosciences, NJ, USA). Counts were processed using R package ‘flowWorkspace’ v4.18.0 [5]. Quality control of the FCS files was carried out using ‘flow_auto_qc’ function in R package ‘flowAI’ [6]. The counts of the stained cells were log transformed and quantified using the ‘gate_flowclust_2d’ function in R package ‘openCyto’ [7], applied on the entire dataset.

Cell abundances of *Prochlorococcus* and *Synechococcus* are reported elsewhere [8] at locations different from those sampled in this study but from the same biogeochemical provinces. Therefore, the abundance of these cells was used to roughly estimate the contribution of cyanobacteria to the total bacterial abundance.

Protein extraction and analysis workflow

The filters were ground into small pieces with a sterile metal spatula after submerging the tubes with the filters into liquid nitrogen. Filter pieces were resuspended in lysis buffer (100mM Tris-HCl pH 7.4, 1% SDS, 150mM NaCl, 1mM DTT, 10mM EDTA) and cells were lysed with five freeze-and-thaw cycles. After centrifugation (20,000x *g* at 4°C for 25 min) the supernatant was transferred into a tube and proteins were co-precipitated with 0.015% deoxycholate and 7% trichloroacetic acid (TCA) on ice for 1h and washed twice with ice-cold acetone. Dried protein pellets were resuspended in 50 mM TEAB buffer (Millipore Sigma, Burlington, MA, USA) and cysteines were reduced and alkylated with 10 mM DTT and 55 mM iodoacetamide (IAA), respectively. To extract proteins associated with the BEVs, we added the sample reducing agent NuPAGE (Invitrogen, Waltham, MA, USA) to 600 μL of purified BEVs at 1X final concentration.

All samples were re-precipitated using 9 times the sample volume of 96% EtOH at -20°C overnight. Pellets were resuspended in 50 mM TEAB, followed by overnight in-solution trypsin (Roche, Basel, Switzerland) digestion (1:100, w/w) at 37°C. TFA was added to the samples at 1% final concentration to terminate trypsin digestion. Samples were desalted using Pierce C18 Tips (ThermoFisher Scientific, MA, USA) according to the manufacturer’s protocol. Prior to the LC-MS analysis, digested peptides were dissolved in 0.1% formic acid and 2% acetonitrile.

Purified tryptic peptides were dissolved in 0.1% formic acid (FA) and measured using a nano-reversed-phase high-performance liquid chromatography (RP-HPLC) system Dionex Ultimate 3000 (ThermoFisher Scientific, MA, USA), coupled with a benchtop Quadrupole Orbitrap mass spectrometer Q-Exactive Plus (ThermoFisher Scientific, MA, USA). Peptides were separated on a PepMap RSLC C18 column (ThermoFisher Scientific, MA, USA) at 55°C with a flow rate of 300 nL/min. The two-hour segmented LC gradient ranged from 5% to 80% buffer B (79.9% acetonitrile, 0.1% FA). Mass spectra were acquired in positive ion mode using a top-15 data-dependent acquisition method. Full MS scans were performed at a resolution of 70,000 (m/z 200), followed by MS/MS scans at a resolution of 17,500. High-energy collisional dissociation (HCD) fragmentation was applied with a normalized collision energy (NCE) of 30%. Dynamic exclusion was set to 60 s.

The mass spectrometry proteomics data have been deposited to the ProteomeXchange Consortium [9] via the PRIDE [10] partner repository.

Figures


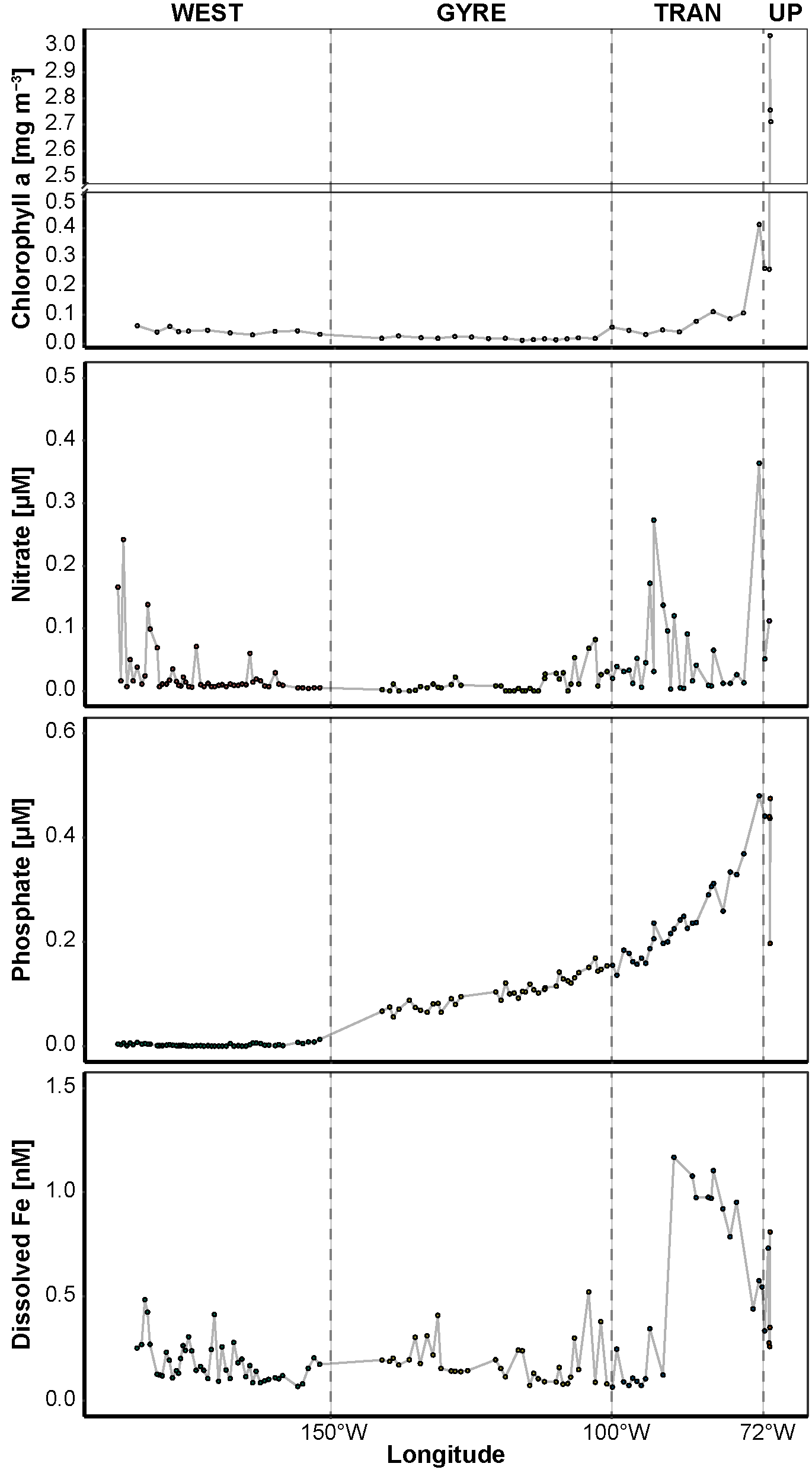


**Fig. S1 –** **Surface water biogeochemical characteristics across the sampling transect.** Spatial grouping of the sampling stations according to four oceanic provinces: ‘UP’ - Chilean coastal upwelling zone; ‘TRAN’ - transition zone between the upwelling and the subtropical gyre; ‘GYRE’ - South Pacific subtropical gyre; ‘WEST’ - westernmost region. Please note the scale differences on the y-axis. Data were previously published elsewhere [11].


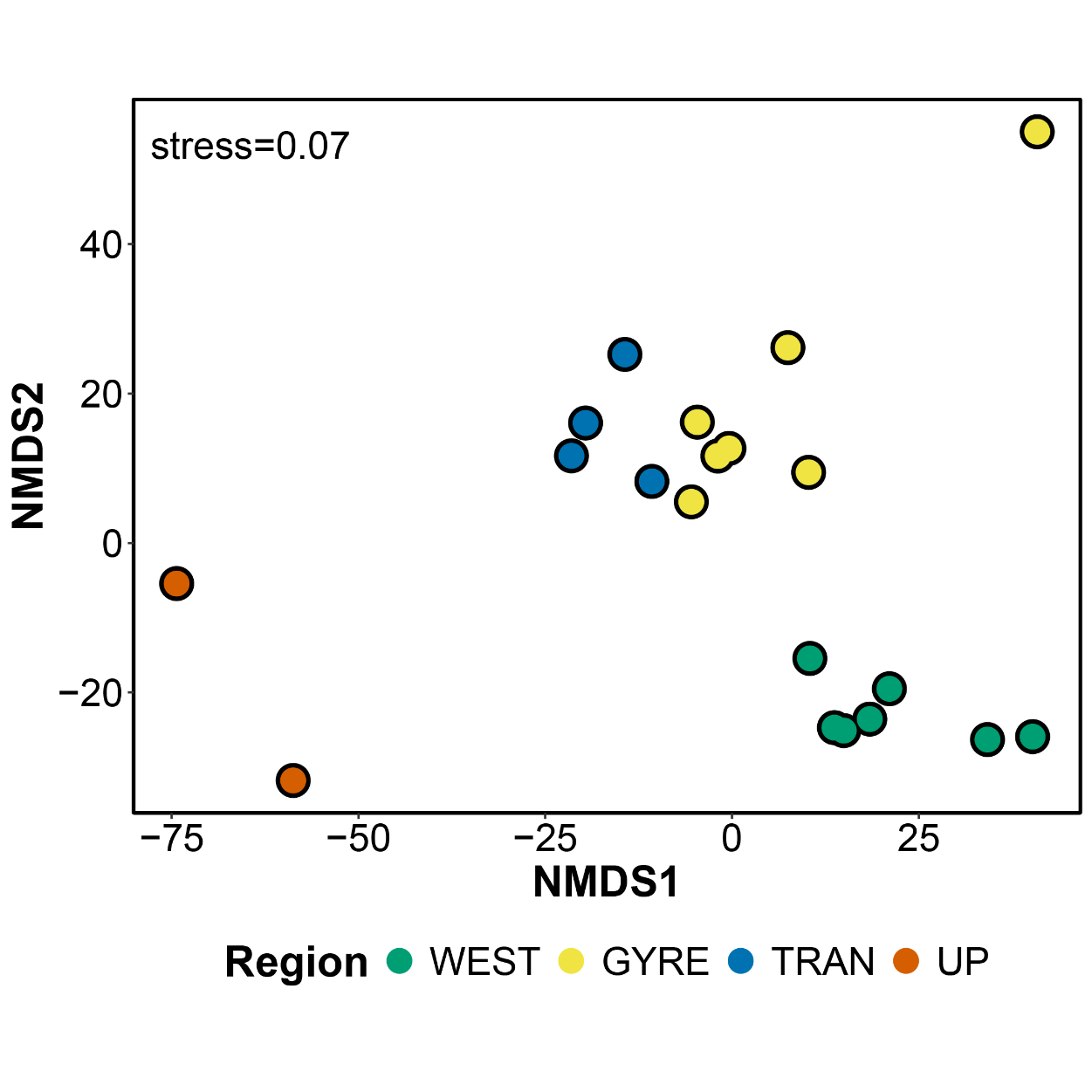


**Fig. S2 – Non-metric Multidimensional Scaling (NMDS) dissimilarities plot of cellular proteomes in each station**. Colors represent the different regions. Dissimilarity was calculated using the Euclidean distance matrix based on log-transformed protein abundances.


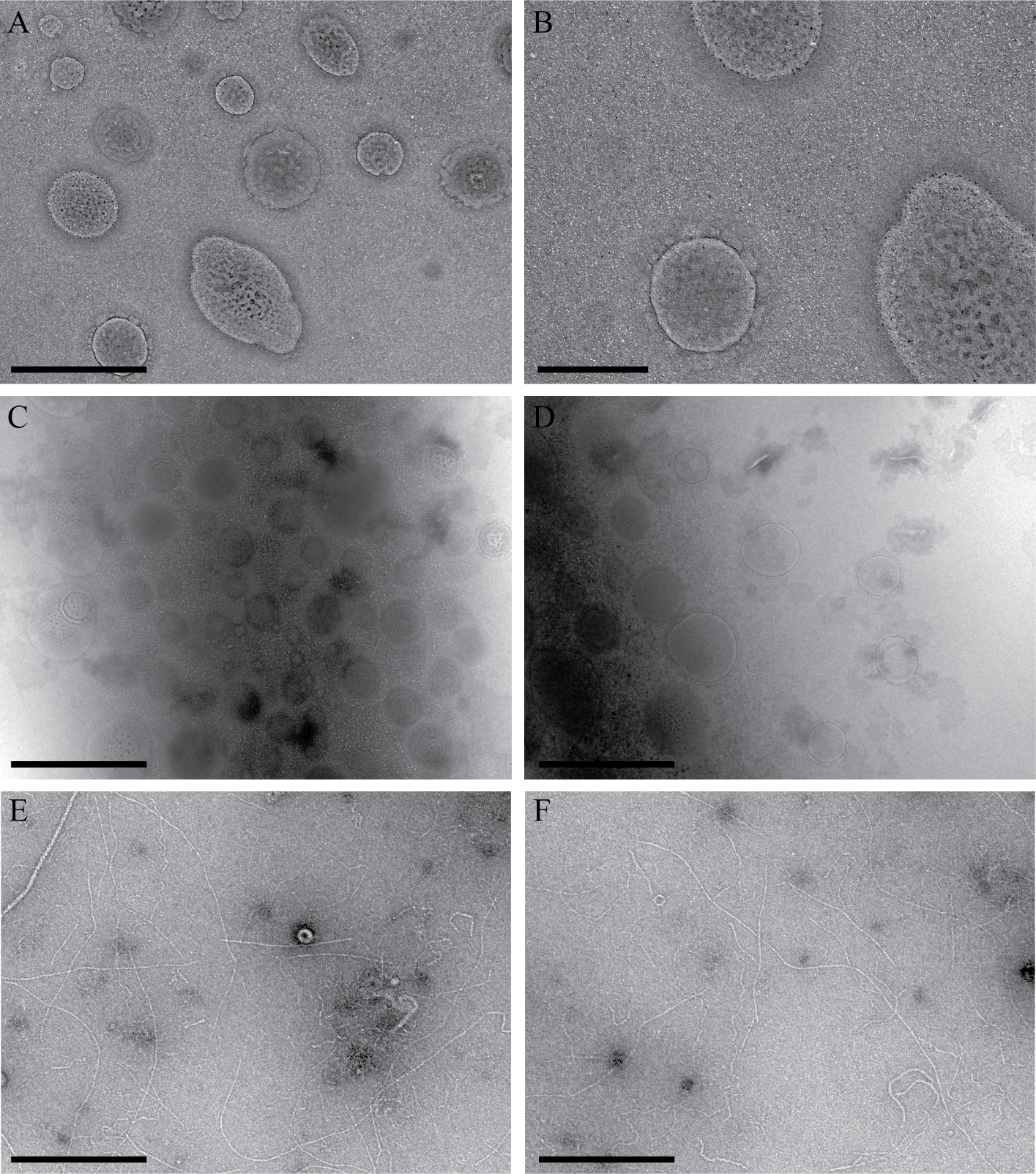


**Fig. S3 – Representative transmission electron microscopy images of BEV-like structures.** Images for the 20-30% density fraction at Station 23 are in panels A and B, with scale bar representing 500 and 200 nm, respectively. Images C and D were taken from 30-40% density fraction at Station 3, with the scale bar representing 500 nm in both. Images E and F were taken from 40-45% density fraction at Station 3, with the scale bar representing 200 nm in both.


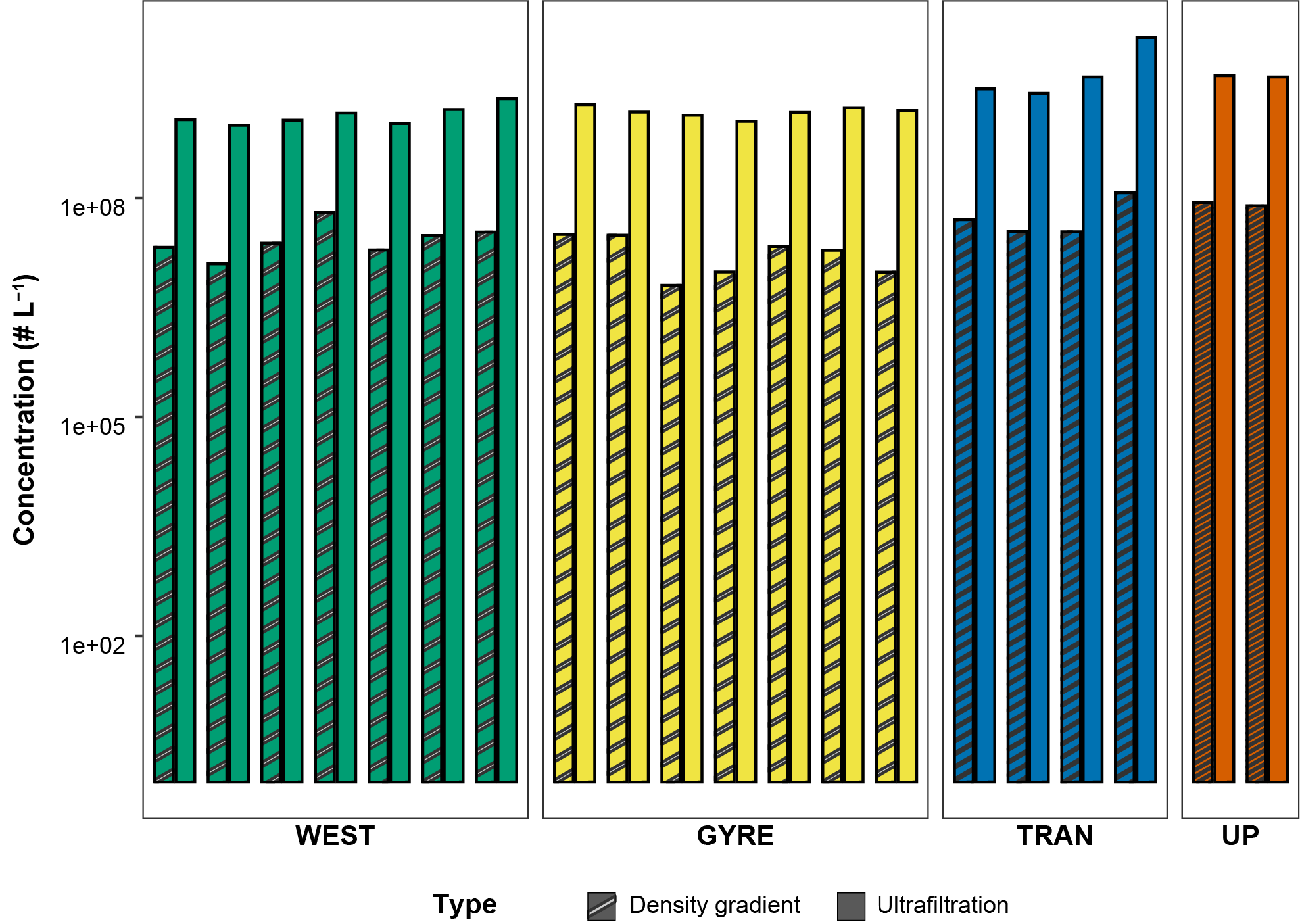


**Fig. S4 – Estimated concentrations of nanoparticles in each station.** The ‘ultrafiltration’ sample represents seawater filtered through 0.22 µm and concentrated using 100 kDa TFF filter. The ‘density gradient’ sample represents the total number of nanoparticles estimated in all different iodixanol density fractions: 10-20% (e.g., small protein complexes, extracellular DNA/RNA), 20-40% (BEVs-enriched fraction), 40-45% (e.g., viral particles, protein aggregates, cell debris).

**
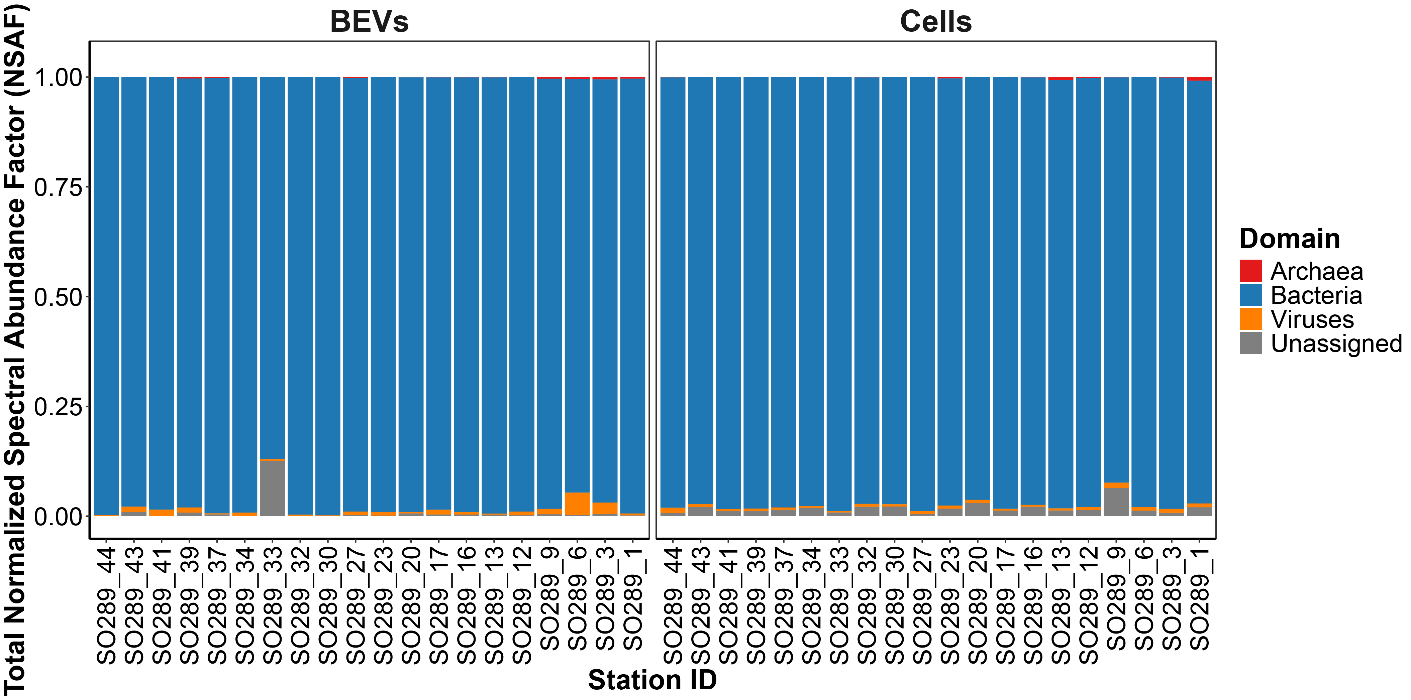
**

**Fig. S5 –Taxonomic composition of metaproteomes across sampling stations.** Stacked barplots show the total Normalized Spectral Abundance Factor (NSAF) summed per sample and colored by Domain.

*
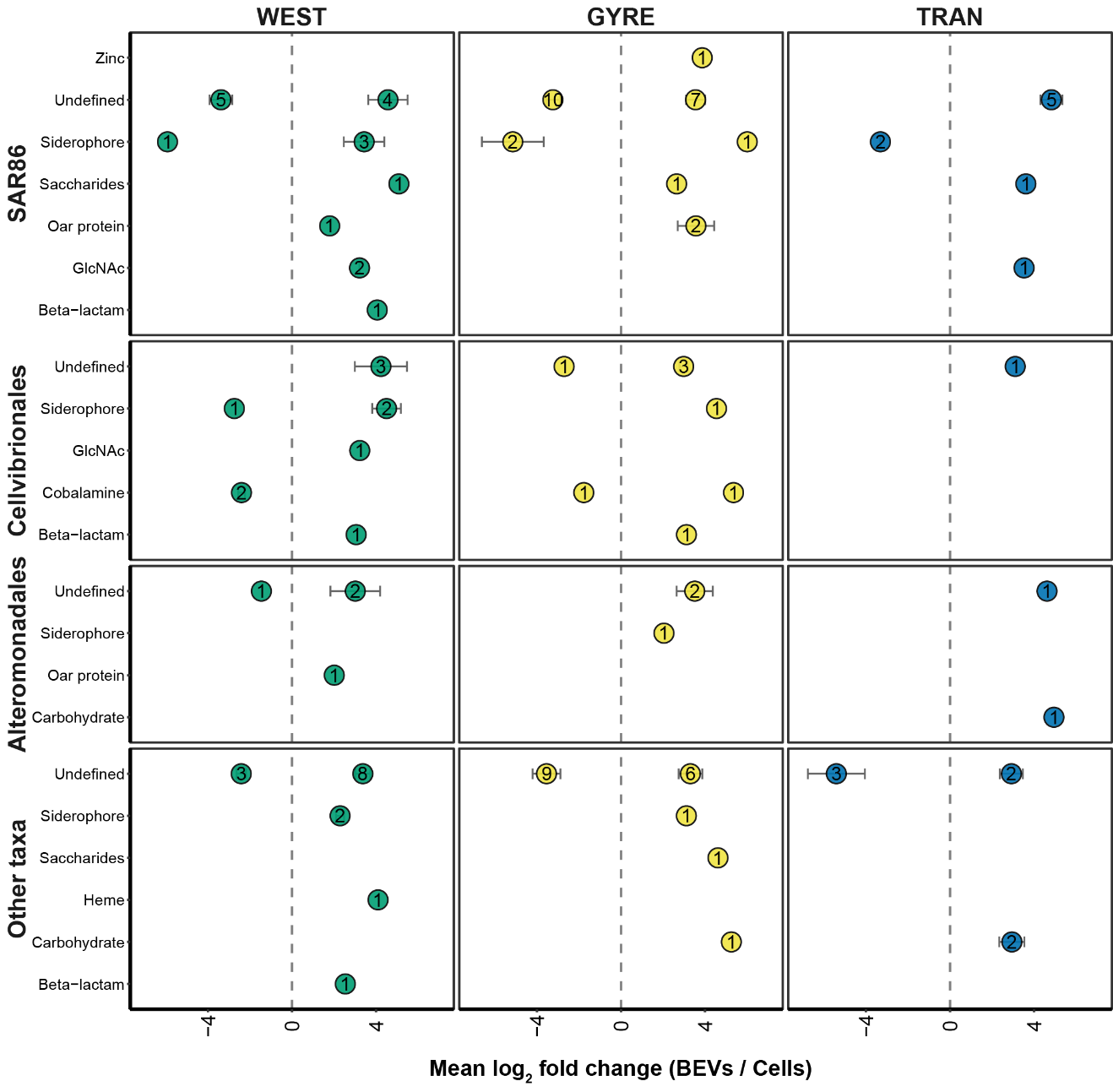
*

**Fig. S6 – Ligand specific TonB-dependent receptors (TBDRs) significantly enriched in BEVs and cellular fractions in different regions**. The mean log₂ fold difference was calculated for each protein group in each fraction. Positive log_2_ fold difference represents significant enrichment in the BEVs fraction, negative mean log_2_ fold difference represents significant enrichment in the cellular fraction. The total number of enriched TBDRs in the BEVs and the cellular fractions is provided in brackets. The TBDRs were grouped according to their predicted ligands and taxonomic affiliation.

Table S1 (separate file). Samples overview.

Table S2 (separate file). Raw protein abundance estimates in each sample.

**Table S3 (separate file).** Results of differential protein abundance analyses between proteomes of each pair of adjacent provinces.

**Table S4 (separate file).** Results of differential protein abundance analyses between BEVs and cellular fractions in each biogegraphical province.

**Table S5 (separate file).** Results of differential abundance analysis of non-cytoplasmic proteins between BEVs and cellular fractions in each province.

**Supplementary References**

1. Browning TJ et al. Nutrient co-limitation at the boundary of an oceanic gyre. *Nature* 2017;**551**:242–246.

2. Van Heukelem L, Thomas CS. Computer-assisted high-performance liquid chromatography method development with applications to the isolation and analysis of phytoplankton pigments. *J Chromatogr A* 2001;**910**:31–49.

3. Mackey MD et al. CHEMTAX - a program for estimating class abundances from chemical markers:application to HPLC measurements of phytoplankton. *Mar Ecol Prog Ser* 1996;**144**:265–283.

4. DiTullio GR et al. Phytoplankton assemblage structure and primary productivity along 170°W in the South Pacific Ocean. *Mar Ecol Prog Ser* 2003;**255**:55–80.

5. Finak G, Jiang M. flowWorkspace: Infrastructure for representing and interacting with gated and ungated cytometry data sets. 2022.

6. Monaco G et al. flowAI: automatic and interactive anomaly discerning tools for flow cytometry data. *Bioinformatics* 2016;**32**:2473–2480.

7. Finak G et al. OpenCyto: an open source infrastructure for scalable, robust, reproducible, and automated, end-to-end flow cytometry data analysis. *PLoS Comput Biol* 2014;**10**:e1003806.

8. Yuan Z et al. Localized nutrient colimitation of phytoplankton growth rates across the subtropical South Pacific Ocean. *Proc Natl Acad Sci U S A* 2025;**122**:e2526930122.

9. Deutsch EW et al. The ProteomeXchange consortium at 10 years: 2023 update. *Nucleic Acids Res* 2023;**51**:D1539–D1548.

10. Perez-Riverol Y et al. The PRIDE database resources in 2022: a hub for mass spectrometry-based proteomics evidences. *Nucleic Acids Res* 2022;**50**:D543–D552.

11. Liu H et al. Patterns of (micro)nutrient limitation across the South Pacific Ocean. *Commun Earth Environ* 2024;**5**:1–9.
